# Validation of a New Resource-Efficient Feeding System for Fattening Pigs Using Increased Crude Fiber Concentrations in Diets: Feed Intake and Ammonia Emissions

**DOI:** 10.3390/ani10030497

**Published:** 2020-03-16

**Authors:** Alexandra Lengling, Bernd Reckels, Cornelia Schwennen, Richard Hölscher, Karl-Heinz Waldmann, Christian Visscher, Wolfgang Büscher

**Affiliations:** 1 Institute of Agricultural Engineering, University of Bonn, 53115 Bonn, Germany; buescher@uni-bonn.de; 2 Institute for Animal Nutrition, University of Veterinary Medicine Hanover, Foundation, 30173 Hanover, Germany; christian.visscher@tiho-hannover.de; 3 Clinic for Swine and Small Ruminants, Forensic Medicine and Ambulatory Service, University of Veterinary Medicine Hanover, Foundation, 30173 Hanover, Germany; Cornelia.Schwennen@tiho-hannover.de (C.S.); Karl-Heinz.Waldmann@tiho-hannover.de (K.-H.W.); 4 Hoelscher + Leuschner GmbH and Co. KG, 48488 Emsbueren, Germany; R.Hoelscher@hl-agrar.de

**Keywords:** feeding technology, roughage-based diet, animal welfare, environmental impacts, liquid feeding system, body composition evaluation, sorting gate, sustainability

## Abstract

**Simple Summary:**

The feeding of fattening pigs and its associated ammonia emissions are current core problems in social debate that affects climate change and sustainability. Feeding methods offer great potential to increase animal welfare and sustainability, and negative impacts on the environment can be reduced. Fattening pigs differ in their performance potential and in their nutrient requirements. A high feed intake capacity can lead to luxury consumption. Diets rich in crude fiber should prevent excess feed intake and cause better nitrogen fixation by microorganisms in the animals’ large intestines. In a pig fattening farm, it was investigated whether and how diets rich in crude fiber can influence feed intake and ammonia emissions. The animals were divided into feeding groups according to their presumed performance potential by ultrasound examinations. Therein, body compositions were evaluated, and feed intake capacity and body weight were automatically recorded. The aim of the study was to enable adapted feeding of the animals by regarding their individual differences in body composition and performance potential. Roughage-based diets had significant influence on feed intake and did not increase ammonia emissions. Based on the results of this study a performance-based control of the feed intake should be made possible.

**Abstract:**

The housing of fattening pigs, their feeding, and the emissions associated with this process are subjects of criticism. In order to reduce emissions and ensure resource efficiency, new paths must be taken; animals must be fed closer to their actual needs. In a pig fattening farm, 655 animals were grouped according their body weight and their body composition, consisting of weight and muscle-fat-ratio, which was determined by ultrasound examinations. The influence of different concentrations of triticale whole plant silage (WPS) (from 2.5% to 10%) on the feed intake capacity (3.88 kg to 2.71 kg (88% dry matter (DM))) of each group and the ability to control it was determined. Ammonia emissions were measured and the pens floor pollution was assessed. The animals could be distinguished significantly from each other by ultrasound examinations. The crude fiber influenced the level of daily feed intake. Ammonia emissions were not negatively influenced and could be partly reduced. There was no negative impact on surface contamination due to the increased use of crude fiber. The amount of daily feed intake was controlled by crude fiber rich diets. If these findings are adapted to certain types of pigs, a reduction of emissions and an increased resource efficiency can be expected.

## 1. Introduction

Livestock production has negative impacts on the environment due to the production of greenhouse gases (GHGs) and ammonia (NH_3_) emissions. Almost 95% of global NH_3_ emissions and 18% of anthropogenic GHG emissions arise due to livestock production [1,2,3]. Besides cattle farming, pig husbandry is the second largest source of NH_3_ emissions in Germany [4]. The main effects of ammonia on the direct environment are eutrophication and acidification of the soil as well as groundwater pollution and indirect nitrous oxide emissions [5,6,7,8]. The fattening pig husbandry has been characterized by an increasing regional concentration particularly in the northwestern regions of Germany over the last few years [9,10,11,12]. This development also leads to negative effects of NH_3_ emissions, which are particularly pronounced in these regions [13].

As part of the protocol to abate acidification, eutrophication, and ground-level ozone and the European Union (EU) Directive 2001/81/EG on the National Emission ceilings, Germany, like all other EU members, has committed itself to reduce NH_3_ emissions by 2030 by 29% (compared to 2005). Since 2010, the maximum of 550 thousand tons of ammonia per year may no longer be exceeded. The limit could not be kept since it was set [14]. Therefore, further activities to reduce NH_3_ emissions are urgently needed.

In the protein metabolism of animals, nitrogen is mainly excreted as urea in the urine (50%) while a small amount is excreted in feces (20%). Only about 30% of the ingested protein is metabolized efficiently and used to gain muscle mass [5]. The enzyme urease produced by bacteria in the feces converts urea in the urine to ammonia, which is then released to the environment [6]. The aim in fattening pigs is a body composition with maximum muscle and minimum fat content [15]. The difficulties arise in the limitations of protein utilization as well as different capacities of feed intake [16], which is influenced, for example, by genetics, gender, age, or the social position of the individual within the group [17]. Different studies have already shown that a reduction of crude protein in the diet of fattening pigs can reduce NH_3_ emissions [18,19,20]. Moreover, the possibility of influencing nitrogen excretion and NH_3_ emission by using crude fiber is well known [21]. In their study, Philippe et al. [22] describe in detail the positive effects of diets rich in crude fiber in fattening pigs. For example, they increased intestinal health and improved the intestine immune system to prevent pathogenic infections. Additionally, increased crude fiber content extends feeding times and functions as enrichment material. Compensatory behavior such as cannibalism due to frustration can be reduced and animal welfare can be improved [23]. It has also been shown that multi-phase feeding strategies with adaption to the changing protein requirements of the animals during the fattening period are preferable to single-phase feeding, with regard to nitrogen excretion of up to 17% [24,25]. However, since current concepts of phase feeding are only based on the average nutrient requirements of the animals, the individual needs are not sufficiently taken into account. Pigs with a high capacity of feed intake consume more protein and energy than they can efficiently utilize. The use of crude fiber is intended to control feed intake and prevent excess intake [26]. Animals with an excess feed intake develop fatter tissue, especially at the end of the fattening period [16]. In addition, excess protein intake leads to increased nitrogen excretion and to an increased release of ammonia. On the other hand, animals below average are not fed sufficiently and are unable to fully achieve their fattening performance potential [27].

Sorting gates are an established technology in large group housing of fattening pigs for many years. This technology enables the automatic selection of for slaughtering by means of optical and mechanical weight determination. Furthermore, it is possible to assign the animals to different feeding areas [28,29]. Until now, the selection and classification of the animals by the sorting gates was based exclusively on the body weight determination. Thus, the animals were divided into “light” and “heavy” animals and could be fed differently according to the average body weight of the group. However, there is still a high potential for saving resources since animals above or below the new average of the two groups (heavy/light) are not supplied efficiently. In order to adapt the feeding of fattening pigs to the current state of the art, a new resource-efficient feeding concept was developed. By introducing new, additional parameters (backfat thickness and the diameter of the *m. longissimus dorsi*), the classification should be refined and the diets should not only be based on average body weight. Feed intake will be used as a quantitative parameter to adjust diets. Furthermore, the different “types of pigs” will be determined by the individual body compositions. Thus, the genetic fattening potential should be used optimally, which is desirable both economically and with regard to resource efficiency. By adapting the diet to the daily measurement data, it should be possible to optimize the protein intake and thus reduce the NH_3_ emissions. For the first time, such new feeding strategy is investigated under barn conditions. In this study, different aspects of the new feeding technology are investigated. On the one hand nutritional aspects like the influence of crude fiber supplemented diets on feed intake of the animals were examined. On the other hand, the influence on NH_3_ emissions and floor pollution was analyzed.

We hypothesize that firstly the feed intake correlates negatively with the crude fiber content in the diet. Secondly, the different “types” of pigs will react differently in their feed intake capacity. Thirdly, the crude fiber does not negatively influence ammonia emissions and floor pollution. Consequently, an individual feeding, resource-efficient, and fattening performance-related feeding concept will be possible.

## 2. Materials and Methods

### 2.1. Animals and Housing

This study was carried out on a pig fattening farm in Lower Saxony, Germany. Three barns were available. Barn I was used to collect data on feed intake and growth performance of the animals under the crude fiber supplemented diet. In addition, ammonia emissions and floor pollution were recorded and investigated. This trial was conducted during a summer fattening period.

**Figure 1:**
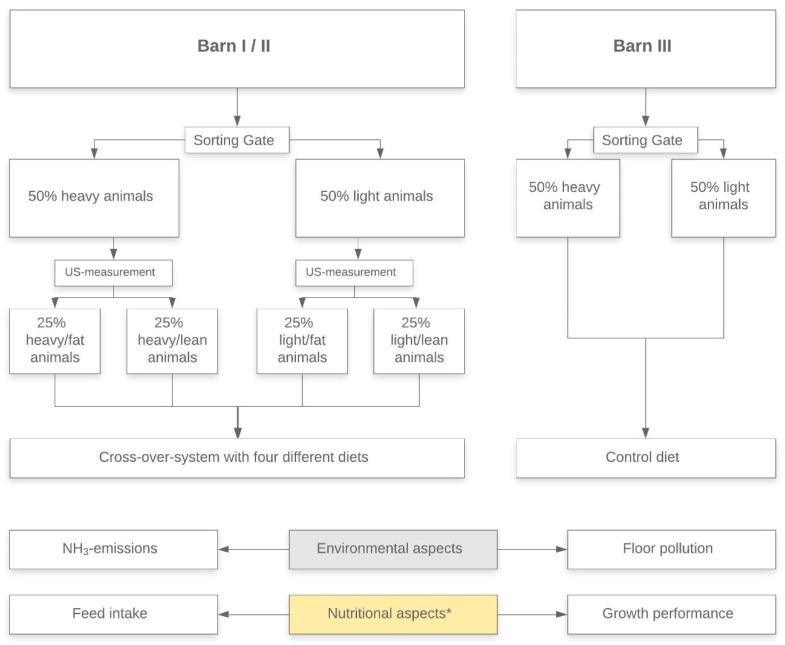
Overview of the measurements done in the different barns. Barn I and II were used for experimental groups with crude fiber supplemented diets. Barn III was used for control group with standard diet (US stands for ultrasound). * Data of nutritional aspects only collected in Barn I.

In Barn II and III measurements during a fattening period with crude fiber supplemented diet (Barn II; same diet as described for Barn I) and during a period with standard diet as control (Barn III) were done in winter. In this trial only data on the environmental aspects were collected, due to technical conditions. Figure 1 gives an overview of the measurements done in Barns I–III.

#### 2.1.1. Barn I

Barn I was subdivided into two pens (I.1 and I.2). It was designed for large group housing with a capacity of up to 656 animals in total and 328 for each pen. In each pen, there was a sorting gate of the company Hoelscher + Leuschner (^®^Hoelscher + Leuschner GmbH and Co. KG, Emsbueren, Germany), which connected the activity and lying areas from two separate feeding areas (feeding area “A” and “B”). In Barn I, there were a total of four feeding areas (two per pen). The sorting gate consisted of an entrance door, a measuring area (including a scale, 3D-camera as well as ear tag recognition) and two exit doors through which the animals can be guided depending on their specific setting. The animals had to pass the sorting gate to reach the feeding areas. The sorting gates allowed for the detection and recording of individual animal data via transponder ear tags. By means of camera technology, which was installed above the sorting gate, each animal was recorded when it entered the gate and the live weight was calculated by measuring the height and width of the animal with the assistance of special software (optiSORT, Hoelscher + Leuschner GmbH and Co. KG, Emsbueren, Germany). In addition, a mechanical weighbridge was integrated in the bottom of the sorting gate so that the current body weight of each animal could be determined. The correlation of optical and mechanical weight determination was verified by Cielejewski et al. [28] and used as the basis for this study. In each feeding area there was a trough, which was filled via a feeding valve (valve 1–6) with a feed in liquid form in different composition.

**Figure 2:**
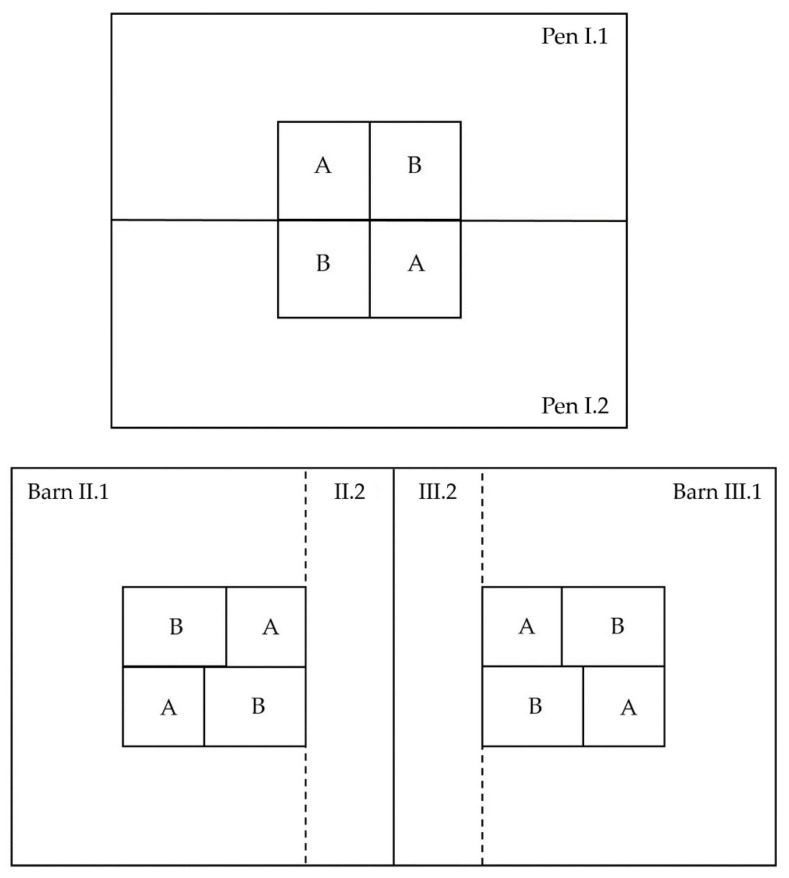
Above: Outline of Barn I with its two pens (I.1 and I.2) and two feeding areas (A and B) per pen. Below: Outline of barn II and III, each subdivided in a bigger (II.1; III.1) and a smaller (II.2; III.2) area and four feeding areas (A and B) per barn.

Barn I had a surface area of 556 m^2^, so approximately 278 m^2^ per pen and a volume of 1780 m^3^ in total. 655 animals were housed in Barn I for the trial. Per animal, there was a usable surface area of >0.8 m^2^. Barn I was used only for measurements under experimental conditions. Approximately 40% of each pen were equipped with a structured plastic slatted floor (Comfi-Floor, Hoelscher + Leuschner GmbH and Co. KG, Emsbueren, Germany) with a void percentage of 3.8%, and 12 mm rectangular openings [30]. The remaining 60% were equipped with concrete slatted floor with a void percentage of 15% and rectangular openings of 17 mm in width. The barn was force ventilated with fresh air supply along the eaves and the decentralized over floor extraction by means of two exhaust fans with a diameter of 1090 mm. Figure 2 shows an outline of Barns I–III.

#### 2.1.2. Barn II and Barn III

Barn II and III were identical in their construction and also designed for large group housing with up to 700 animals per barn. Each barn was subdivided in a bigger area (II.1 or III.1) and a smaller area (II.2 or III.2). Area II.1 and III.1 had a surface of 452 m^2^ and a volume of approximately 1650 m^3^ each. Area II.2 and III.2 had a surface of 133 m^2^ and a volume of 486 m^3^. The access to the smaller areas was closed at the beginning of the fattening period and was opened after the first quarter of the period to provide more space to the animals. Per animal there was a usable surface area of >0.75 m^2^. Each barn was equipped with two sorting gates that connected the activity and lying areas from separated feeding areas. Barn II was used for measurements under experimental conditions, while Barn III was used as the control barn. Approximately 60% of the surface area were equipped with a structured concrete slatted floor with a void percentage of 6% and rectangular openings of 17 mm in width. The other 40% were equipped with concrete slatted floor, as described for Barn I. Moreover, as described above for Barn I, each barn was force ventilated with four exhaust fans with 980 mm diameter (Figure 2).

#### 2.1.3. Animals

In Barn I, the 655 piglets came from a farm in Brandenburg, Germany and were crossbred products of a Danzucht and Bundes Hybrid Zucht Program (BHZP; German Federal Hybrid Breeding Program). The animals all came from one litter group. The males were castrated as suckling pigs and their average body weight at the time of housing was 25 kg. The animals were randomized into two pens so that two identical groups were formed by size and weight. Randomization was ensured by the fact that the two pens were not separated from each other by the otherwise closed connecting doors before data collection and examinations began. Dividing the animals into two pens for maximum comparability was controlled and could be confirmed by the almost identical mean body mass of the animals in the groups.

In Barn II and III, 700 piglets with the same genetics as used in Barn I were housed in for the experimental group and the control group, respectively. The fattening period of the control group started at the beginning of November 2018, while the period of the experimental group started four weeks later. Due to the large number of piglets needed for both groups, it was not possible to house in both groups at the same time. The average body weight at the beginning was about 40 kg for the control group and 25 kg for the experimental group.

### 2.2. Feeding

#### 2.2.1. Feeding Groups

The experimental groups were divided into four feeding groups. This was done by means of ultrasound examination (see Section 2.2.2) of each animal (Ø bodyweight 50 kg; standard deviation ± 6.42 kg). The groups were divided according to the parameters of body weight and the ratio between back fat thickness and the diameter of *Musculus longissimus dorsi*. Based on the weighing data, all animals were subdivided into “light” (Pen I.1) and “heavy” (Pen I.2) with roughly equal proportions. In addition, all animals were categorized as “fat” and “lean” based on the ultrasound data and the calculated back fat/muscle ratio (see Figure 1). The categorization was carried out for both the light and heavy animals, so that one group of light animals with a low back fat/muscle ratio (“light lean (LL)”) and a second group of light animals with a high back fat/muscle ratio (“light fat (LF)”) originated. The heavy animals were grouped as “heavy lean (HL)” and “heavy fat (HF)”, respectively. The division of the animals in the subgroups and the associated feeding areas was ensured by the recognition of the individual transponder ear tags and the sorting gates. The gender was initially not taken into account in the division.

#### 2.2.2. Ultrasound Examination

**Figure 3:**
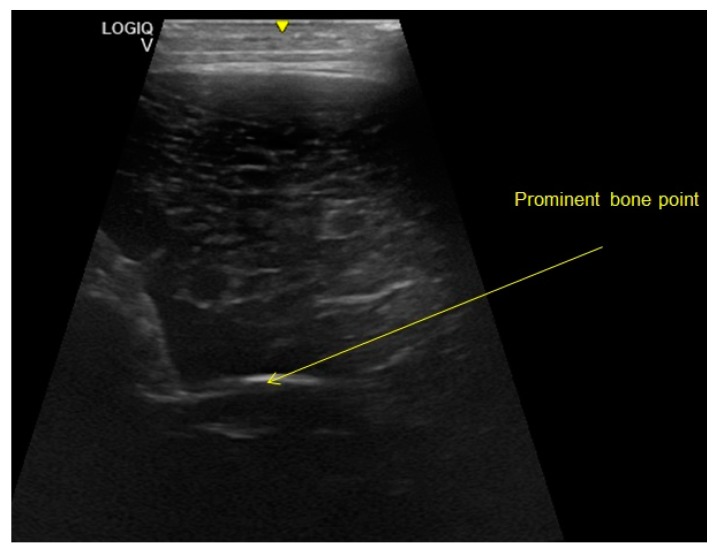
Ultrasound image taken in these examinations in order to illustrate the prominent bone point (photo: ^©^Reckels, University of Veterinary Medicine, Hanover).

The ultrasound examination was performed with the LOGIQ® V2 device (GE Healthcare, Little Chalfont, UK). The measurement was made in the exit gate after the feed intake of the animals. The measuring point was chosen at the side of the spine at the height of the last rib on the left side of the body. This measuring point was based on the P2 measuring point [31]. In younger animals, 6–8 cm of the greatest possible muscle thickness running laterally along the back line cannot be detected [32] and so the point was adapted to the body mass and body condition of the growing animals. In order to be able to judge the same point with each measurement, a prominent bone point was used that was anatomically visible on the ultrasound images of the 655 evaluated pigs (Figure 3).

**Figure 4:**
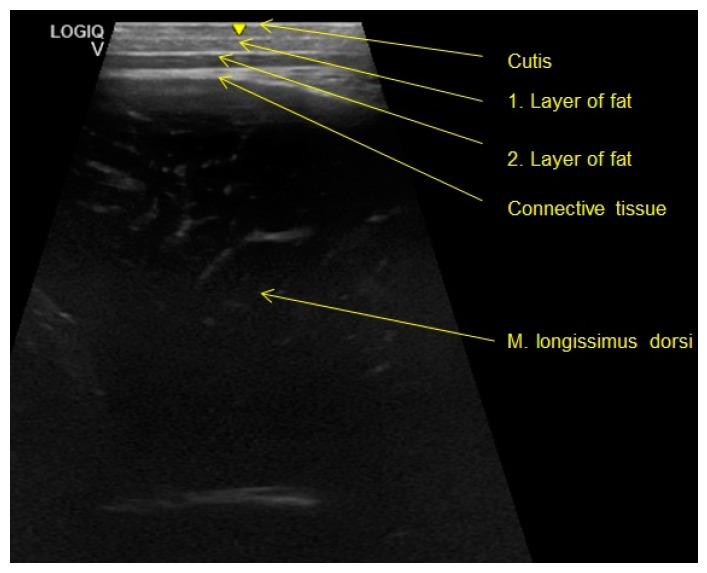
Ultrasound image taken in these examinations in order to illustrate the layers mentioned (photo: ^©^Reckels, University of Veterinary Medicine, Hanover).

The back fat and muscle thickness was determined on all animals in the compartments. The measurement was performed on the *longissimus dorsi* muscle and recorded the back fat thickness, which consists of the skin, the first layer of fat (*subcutis*) and the second layer of fat (interfacial fat layer) as well as the connective tissue, which is located above the *longissimus dorsi* muscle and the muscle thickness of said muscle (Figure 4) [33].

#### 2.2.3. Feeding Technology and Feeding Components

The fattening pigs on the farm are fed exclusively with a liquid feeding system (^®^Hoelscher + Leuschner GmbH and Co. KG, Emsbueren, Germany). The liquid feed for the fattening pigs was composed of six different components (complete feed, supplementary feed, and feed material) in different percentage proportions. Due to the storage on the test farm, however, it was only possible to use five different components at the same time. The complete feed, “CF1”, as well as the supplementary feed, “SF1”, “SF2”, and “Soybean oil”, were purchased from a German feed company and were used as the sole component and part of the compound feed. Corn-Cob-Mix (CCM) and Triticale whole-plant silage (WPS) were used as the farm’s own components. The diets were designed according to the demand of the animals and components were used according to the ingredients. Table 1 shows the chemical compositions of the used components.

The water supply was additionally provided by an open drinking trough and 30 drinking nipples per barn. In addition, the animals were granted access to roughage through a suspended hay rack. However, since the hay was laced, the hay bales had to be renewed once a week, and the roughage could not be seen as a feed but as additional enrichment material.

#### 2.2.4. Feeding Scheme and Diets

The feed was offered to all feeding groups *ad libitum* throughout the fattening period. The composition of the diets was adapted two times in the course of the fattening period in order to meet the demands of energy and nutrient requirements [35] (see Table S1 and Table S2). The sorting gates automatically determined the average body mass of the feeding group and individually graded the animals. Based on this subdivision, individual animals were sorted out into the appropriate feeding area. All feeding compositions were designed on an equal amount of energy and nitrogen, yet the amount of crude fiber significantly differed. The diets offered to the four different groups were changed every six days. The average daily feed intake per animal was calculated by the installed feeding technique. Table 2 shows the diets for an average bodyweight of 70 kg in the experimental groups.

Table 3 shows the diets for an average bodyweight of 70 kg of the control group.

Table 4 shows the nutrient contents of all diets in Barns I–III. The basis for the calculation were the values from Table 1. The Triticale WPS and the CCM were analyzed at the Institute for Animal Nutrition in Hanover [34].

### 2.3. Emission Measurement Techniques

#### 2.3.1. Gas Sampling and Emissions Calculation

The ammonia concentrations were measured for the control group and both experimental groups inside and outside the barns. For the measurement photoacoustic-infrared-spectroscopy (PAS) was used. Barn I was equipped with a Multi-Gas-Monitor Innova 1314 and a Multipoint-Sampler 1309 (LumaSense Technologies A/S, Ballerup, Denmark), while Barn II and III were equipped with a Multi-Gas-Monitor Innova 1412i and a Multipoint-Sampler 1409. The measurement was carried out using the methodology as described by [36,37]. The measurement of fresh and exhaust air was done continuously for the control group and experimental groups, respectively. In each barn, one air sampling point outside and inside were installed. The sample points for fresh air were installed at the air inlets at the eaves. The sample points for exhaust air were installed below an exhaust fan inside every barn.

The emissions (E_Gas_) were calculated using the following equation:

E_Gas_ = V ∗ (C_in_ − C_out_) (1)

where V is the hourly ventilation rate (m^3^ h^−1^) based on the average of 12 values per hour and C_in_ and C_out_ are the hourly gas concentrations (gm^3^) inside and outside the barns based on 15–18 values per hour. The average daily emissions were calculated and expressed as g d^−1^ per livestock unit (LU; equal to 500 kg body weight).

#### 2.3.2. Ventilation Rate, Temperature, and Relative Humidity

The ventilation rate was estimated using means that measured fans (Reventa GmbH, Horstmar, Germany). The measuring fans were calibrated by the manufacturer. Below each exhaust fan in each exhaust chimney, a measuring fan was installed. The measurement data were recorded by Almemo 2590 data loggers (Ahlborn Mess- und Regelungstechnik GmbH, Holzkirchen, Germany) every five minutes.

At every measuring point for gas concentration in the exhaust air, the temperature and relative humidity were recorded with data loggers Testo 174 H (Testo SE and Co. KGaA, Lenzkirch, Germany) every five minutes. One Testo data logger was installed outside to record external climatic conditions.

#### 2.3.3. Emission Data Analysis

For analysis of the emission situation, the fattening periods were divided into sections. For each group (Barn I = experimental group summer; Barn II = experimental group winter; Barn III = control group) 10 measuring days were evaluated during the middle of the fattening period (average weight section 75–85 kg) and during the final part of fattening period (average weight section 105–115 kg), respectively. Additionally, a period of 30 measuring days from the middle to the final of the fattening period (average weight section 70–110 kg) was analyzed. Each measuring day was based on n = 415 and n = 350 (for Barn I and II/III) single values for gas concentration. For ventilation rates, temperature, and humidity measurements, n = 288 values per day were analyzed.

### 2.4. Evaluation of Floor Pollution

To investigate whether the crude fiber supplemented diet influenced floor contamination, an evaluation for the experimental group during summer in Barn I was conducted and modified according to Austermann [36] and Ebertz et al. [38]. The floor pollution of a control group in Barn III was evaluated. For each barn, the total surface area was determined. Despite the presence of different types of slatted floors, the total void percentage in the two barns did not significantly differ from each other (10.8% Barn I; 9.6% Barn III), so that comparability was possible.

The surface area was subdivided into score areas according to different floor elements. Due to the different types of slatted floor, the score areas differed in size. The percentage of each score area was calculated as a percentage of the total surface area, so that the total percentage of pollution could be calculated by the end. Each score area was individually evaluated. This evaluation concerned the pollution of surface areas (0 = clean and dry; 1 = wet; 2 = polluted; 3 = wet and polluted; 4 = muddy) and occlusion of slats (0 = 0%–25% blocked; 1 = 26%–50% blocked; 2 = 51%–75% blocked; 3 = 76%–100% blocked). For the experimental group, a total of six evaluations were carried out (30, 50, 65, 85, 100, and 120 kg average body weight). Due to external circumstances, only four evaluations could be carried out in the control group (30, 40, 65, and 85 kg average body weight). In all cases, the evaluation was done by the same person at a three-week interval. This enabled an evaluation of floor contamination that was as evenly distributed as possible over the entire fattening period for both groups.

### 2.5. Statistical Analysis

The statistical analysis was done using IBM SPSS Statistics 24 and the statistical software package from SAS, Version 7.1 (SAS Inst., Cary, NC, USA). All measured data were analyzed descriptively by sample size, mean values, confidence interval, standard deviation, minimum, and maximum (environmental data). For the environmental data, the Kolmogorov–Smirnov test was used to test for normal distribution. If normal distribution was present, a simple variance analysis was used to determine significance. Otherwise the Kruskal–Wallis test was used. All charts were created with Microsoft (MS) Office Excel 2016. The group comparisons were performed by one-way analysis of variance (ANOVA) for independent samples. The sum of the mean daily feed intake per animal was compared in four different groups (HF/HL/LF/LL), which were constantly divided, under the different diets. The mean values were compared with each other. In general, the Ryan–Einot–Gabriel–Welsch multiple-range test (REGWQ) was used for multiple pairwise means comparisons between the four groups. All statements of statistical significance were based on *p* < 0.05.

## 3. Results

### 3.1. Animal Based Data During the Experiment

#### 3.1.1. Body Composition during Group Building

Table 5 shows the body mass (kg), gender, back fat thickness (cm), diameter of the *Musculus longissimus dorsi* (cm), and the ratio of the back fat and muscle of the fattening pigs at the grouping time.

Significant differences can be seen for “body mass” between the “light (LL/FF)” and the heavy (HL/HF)” groups (Table 5). Additionally, there is significant difference between the heavy group (HL to HF). At the parameter of back fat thickness, there are significant differences between all four groups. The muscle diameter is nearly equal in the heavy groups (HF/HL) but there are significances between the animals of LF and LL and both heavy groups. Taking the ratio between back fat and muscle, there are significant differences between the “fat” groups (HF/LF) and the “lean” groups (HL/LL).

#### 3.1.2. Feed Intake

Table 6 shows the average feed intake of four different diets for four different groups per animal and day. Every diet was fed for 18 days in total.

Comparing diet 2 and diet 4, there are significant differences in daily feed intake in the LF and LL group. For the feed intake of the HL group, there are significant differences between diet 1 and all other diets. In the HF group, there are no significant differences but a tendency of reduction can be seen in the comparison of diet 1 and 4. Differences can also be seen between the feeding groups with regard to feed intake; lean animals tend to have a lower feed intake than fat animals for almost all diets.

### 3.2. Environmental Aspects

#### 3.2.1. Climatic Conditions

Table 7 shows the average of ventilation rates per livestock unit; internal and external temperatures; and internal and external relative humidity measured during the different weight sections for the control and experimental groups, respectively.

Compared to CD, significant differences could be determined for both CFD_W_ and CFD_S_ with regard to climatic conditions. However, due to the different seasons during the experiments, the differences between CD and CFD_S_ are more pronounced, especially for ventilation rate and external temperature. A comparative analysis of the data is only possible to a limited extent. Nevertheless, for completeness, the results of CFD_S_ are presented in order to be able to compare and discuss them with results reported in other studies. For the weight section, 70–110 kg, the measured external temperature in CFD_W_ was 2.5 °C higher than in CD. In comparison, the average external temperature in CFD_S_ was 15.5 °C higher than in CD. According to the measured external temperatures, the ventilation rate per LU for CFD_S_ was, on average, 303.4 units higher than in CFD_W_ and CD. In contrast, CD and CFD_W_ only differed by 21.5 m^3^ h^−1^ LU^−1^. Due to the considerably higher ventilation rate during CFD_S_, the internal temperature was 3.5 °C higher than in CD. For CD and CFD_W_, a difference in mean internal temperature of only 0.6 °C was noticed. The relative humidity inside during CFD_S_ was 12 units below those measured during CD. For CD and CFD_W_, the difference was −5.5 units. Relative humidity measured outside during CFD_S_ was around 26.5 units. During CFD_W_ it was eight units lower than during CD. The results for the separate evaluation of middle- and end-part of the fattening period can be seen in Table 7.

#### 3.2.2. NH_3_ Concentration and Emissions

**Figure 5:**
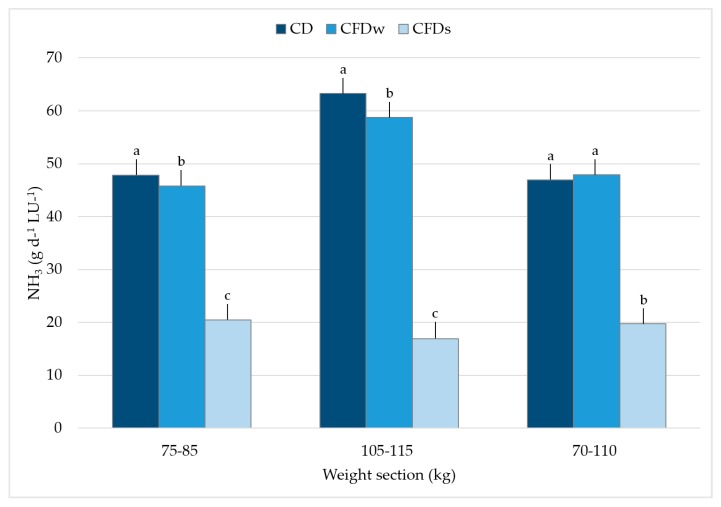
Ammonia emissions per day and livestock unit as influenced by the diet (CD = control diet; CFD_W_ = crude fiber diet winter; CFD_S_ = crude fiber diet summer) according to the weight section. Weight section 75–85 kg and 105–115 kg had an average of 10 days. Weight section 70–110 kg had an average of 30 days. a, b, and c significantly differ from each other.

Figure 5 shows the average ammonia emissions in g per day and the LU for the three selected weight sections per animal group.

For the 75–85 kg weight section, emissions for CD, CFD_W_, and CFD_S_ were 47.8 ± 8.4, 45.8 ± 10.5, and 20.4 ± 5.5 g d^−1^ LU^−1^, for the 105–115 kg weight section, 63.3 ± 20.2, 58.7 ± 25.1, and 16.9 ± 10.8 g d^–1^ LU^–1^; and for the 70–110 kg weight section 47.0 ± 9.9, 48.0 ± 9.9, and 19.8 ± 9.0 g d^−1^ LU^−1^. For all three weight sections, the ammonia emissions for CFD_S_ significantly differed from the CD and CFD_W_ (*p* < 0.05). During winter, increased crude fiber content in the diet resulted in 4.2% and 7.3% less ammonia emissions for weight sections 75–85 kg and 105–115 kg compared to CD. Both differences are significant (*p* < 0.05). Nevertheless, for the weight section 70–110 kg, the average ammonia emissions were 2.1% higher in CFD_W_ than in CD, which is not a significant difference (*p* > 0.05). The ammonia emissions were 55.5%, 71.2%, and 58.8% lower (weight sections 75–85, 105–115, and 70–110 kg) in CFD_S_ compared to CFD_W_.

**Figure 6:**
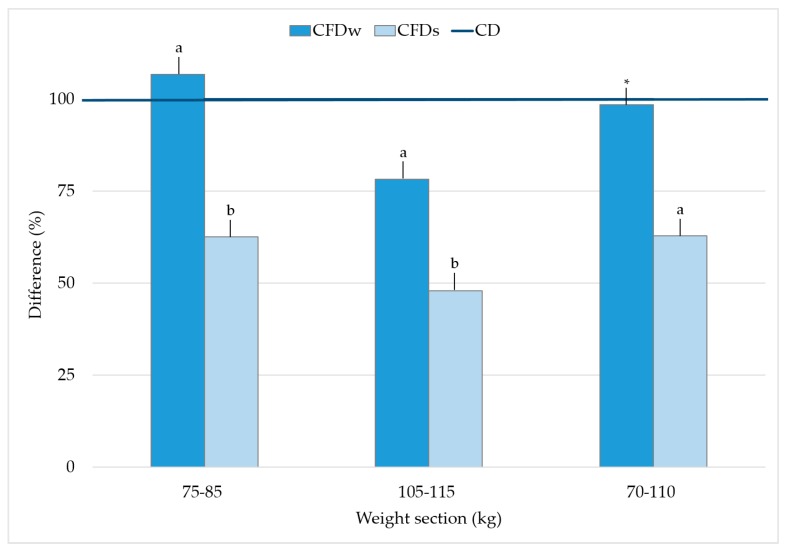
Percentual difference of mean ammonia concentrations measured in the exhaust air for CFD_W_ and CFD_S_ in relation to CD (defined as 100%). a and b significantly differ from CD; * no significant difference from CD.

The ammonia concentrations measured in the exhaust air for weight section 75–85 kg was 6.8% higher for CFD_W_ than for CD. In contrast, the concentrations in the last part of the fattening period (105–115 kg) were 21.8% lower for CFD_W_ than for CD. During both sections of the fattening period, the differences were significant (*p* < 0.05). On average, the ammonia concentrations of the weight section 70–110 kg were reduced by 1.5% for CFD_W_ compared to CD, which was not significant. The ammonia concentrations of CFD_S_ were significantly lower than those of CD (37.4%, 52.0%, and 37.1% for weight section 75–85 kg, 105–115 kg, and 70–110 kg). The results are shown in Figure 6.

#### 3.2.3. Pollution of Surface Area

In general, the formation of functional areas by the animals could be reconstructed during the evaluation of surface pollution. The feeding areas and areas next to them could be described as “dry and clean”. These were used as lying and activity areas. In contrast, the areas near the outer walls of the barns were used for excreting feces and mostly described as “polluted” (see Figure S1).

**Figure 7:**
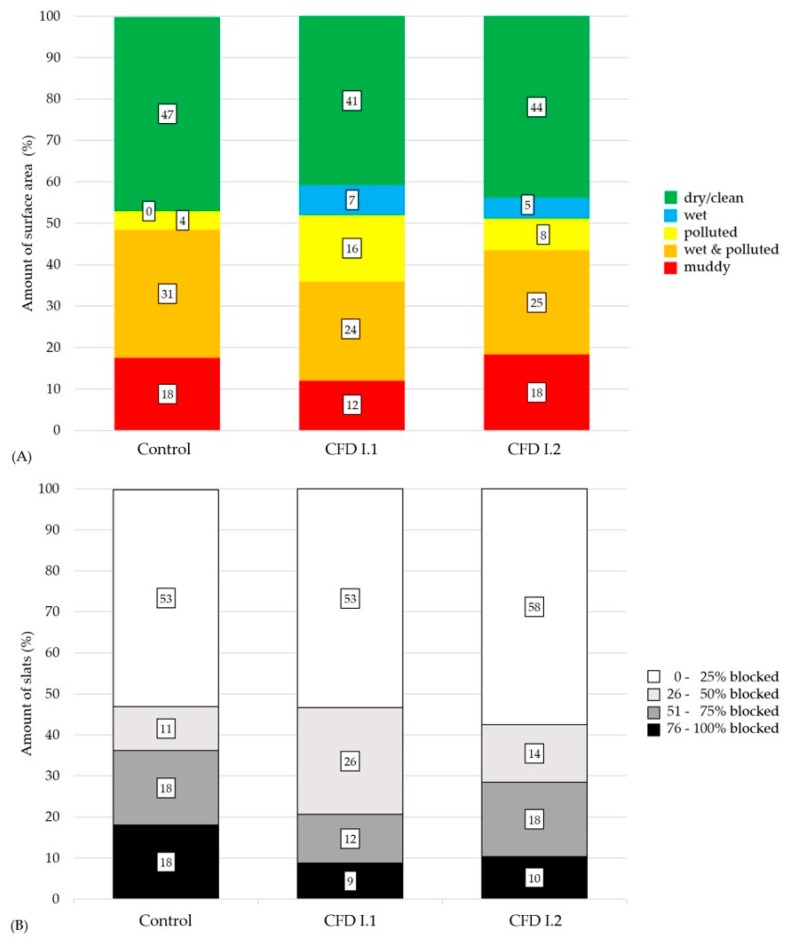
Mean amount of polluted surface area (**A**) and occluded slats (**B**) in % for the control and experimental groups (pen I.1 and I.2). Figure follows [38].

For the assessment of surface contamination, the results of four days of evaluation per animal group were compared (the first four evaluation days were from the average body weight section 30–85 kg). On average, the clean surface area was 47% ± 7.4%; the polluted surface area was 53% ± 5.2% in the control group. In the experimental group, the amount of clean surface area was 41% ± 8.6% and 44% ± 7.6%. For the polluted surface area it was 59% ± 8.5% and 56% ± 7.2% (Pen I.1 and I.2). A contaminated area of 0.4 m^2^ per animal could be determined for CD and CFD, respectively. No significant difference was observed between groups (*p* > 0.05). Figure 7A shows the mean percentage of surface area within the five evaluation categories. No significant differences could be found between the groups for the different categories (*p* > 0.05).

The results of the slat occlusion evaluation are suitable for the results of surface contamination in all three groups. On average, the number of slats blocked 0%–25% and did not significantly differ within groups (53% ± 7.4%, 53% ± 9.4%, and 58% ± 12.8% for control, CFD I.1 and CFD I.2; *p* > 0.05). The number of slats blocked 26%–50% was significantly higher in CFD I.1 (26% ± 3.4% compared to 11% ± 8.8% for control and 14% ± 6.0% for CFD I.2). For all other categories, no significant difference was found. The detailed amounts of slat occlusion are shown in Figure 7B.

## 4. Discussion

### 4.1. Heterogeneity of Animals and Classification According to Genetic Performance Potential

Although all animals up to 50 kg were kept and fattened under the same conditions, there is a clear heterogeneity in the whole group. A significant difference was the body weight (HF/HL to LF/LL) between the heavy and light animals. In order to ensure an energy and protein adapted diet and avoid oversupply, the heavy animals must be separated from the light animals and fed differently. The composition of the mass growth of a fattening pig changes in the course of its development. With a body mass of 60–70 kg, the protein content of the body mass decreases, and the fat content continues to increase [39,40]. In addition, the composition of biomass is determined by genetics and gender. According to Kirchgeßner [41], genetic protein uptake has a significantly higher influence on the protein approach than the different protein and energy supply. These sources were confirmed by the experiment. Table 5 shows that approximately 60% of lean animals (HL/LL) were female. Conversely, about 60% of the fatter animals (HF/LF) were castrated boars.

This potential is reflected in the fat-muscle ratio. The different animal species can be significantly separated by recording the fat-muscle ratio (HF/LF to HL/LL). However, fattening pigs with a high feed intake capacity and a low genetic performance potential automatically absorbs more energy and protein, which they cannot use efficiently. As a result, they develop more fatty tissue and excrete more nitrogen [42]. Therefore, the starting point for resource-efficient feeding is the classification of fattening pigs into four different subgroups according to their presumed genetic performance potential (HF/HL/LF/LL) (Figure 1).

### 4.2. Influence of a Feed Rich in Crude Fiber on Daily Feed Intake Capacity

The aim of the study was to classify the pigs according to their suspected genetic potential and see whether they react differently to the increased crude fiber content in relation to their daily feed intake. Resource efficiency can only be guaranteed if fattening pigs are fed according to their genetic protein accretion potential and oversupply is prevented. One hypothesis was to control feed intake by using a structured crude fiber source. Looking at the average daily feed intake of the animal, the daily feed intake for all four groups (HF/HL/LF/LL) had an increased use of Triticale-WPS and ultimatley decreased. The difference of the average daily feed intake in the “heavy-lean” animals (HL) differed significantly between the diet with the lowest crude fiber content (diet 1) and the diet with the highest crude fiber content (diet 4). In both groups of light animals (LF/LL), a significant difference between the second and the fourth diet was seen.

However, in all four groups, there is a clear tendency to see a decrease in daily feed intake with increased use of crude fiber. The saturation of pigs is basically divided into mechanical and chemical saturation [43]. Mechanical saturation is caused by the filling of the stomach. Stretch receptors transmit a signal via vagus fibers to the hypothalamus, with a neural network regulating saturation [16,44]. With regard to the saturation effect, WPSs are important as a source of crude fiber. Due to the high proportion of acidic detergent fibers (ADF) and hemicellulose [27], as well as the high proportion of bacterially fermentable substances [45], WPS contributes to saturation. Another important contribution is the high-water retention capacity of feedstuffs containing crude fibers [46,47,48], which supports mechanical saturation. Heinritz et al. have shown that a feed rich in crude fiber has a significant influence on an animal’s microbiome. It can therefore be assumed that the proportion of Lactobacilli and Bifidobacteria has increased due to an increased fiber content [49]. There is evidence that SCFAs produced by the microbiota interact with enteroendocrine host cells (e.g., L-cells) by modulating the G-protein coupled receptor signal (GPR41, GPR43), which affects the production of glucose homeostasis modulators such as peptide YY (PYY) and glucagon-like peptide (GLP)-1 [50]. Tyrosine (PYY) and glucagon-like peptide-1 (GLP-1) from entero-endocrine cells. Both hormones influence saturation via an effect in the brain and on the “ileal brake”. The presented results support these results.

### 4.3. Environmental Aspects

For CFD_W_, significantly lower ammonia emissions were observed for weight sections 75–85 kg and 105–115 kg, compared to CD. No significant difference was found for weight section 70–110 kg between CD and CFD_W_. The highest difference between CD and CFD_W_ was found for the final weight section. As mentioned above, the protein requirement of fattening pigs decreases at the end of the fattening period [16]. At the same time, there is an increase in feed intake [27]. Fattening pigs with a high capacity of feed intake excrete more nitrogen, as they cannot efficiently utilize all of the absorbed protein [42]. This is particularly noticeable at the end of a fattening period. Consequently, in the presented feeding, the greatest saving potential for ammonia emissions was the last part of the fattening period. The presented results of ammonia emissions support this theory. As presented, feed intake decreases with increasing crude fiber content in the diet. At the same time, an increased fiber content supports bacterial growth in the animals’ intestinal tract. As a result, more nitrogen can be incorporated in bacterial protein, so that less nitrogen is excreted with urine. This can reduce ammonia emissions [16,42]. In the presented study, avoiding luxury consumption and increased nitrogen fixation in the intestine may have led to a reduction in ammonia emissions. However, the last point was not investigated and can only be assumed.

The ammonia emissions determined for CD and CFD_W_ are comparable to those described by other authors. Gallmann [51] reviewed ammonia emissions of 41–160 g d^−1^ LU^−1^ while Philippe et al. [24] reported an average value of 68.5 g d^−1^LU^−1^. Demmers et al. [52] reported an average value of 51 g d^−1^ LU^−1^ during the winter period. Compared with the reported values in the literature, the measured ammonia emissions in this study are at a lower level.

For the experimental period during the summer, significantly lower ammonia emissions were observed for all weight sections compared to CD and CFD_W_. The presented values are markedly lower than described in the literature. Various studies have reported on the correlation between ventilation rates, exhaust air concentrations, and emission rates from animal houses [5,53,54]. For an approximately constant room temperature in the animal area, higher ventilation rates are measured in summer because of higher external temperatures. Due to a higher air exchange rate, ammonia emissions increase in summer months [36,52]. However, concentrations decrease inside due to a dilution effect [55].

Other authors reported contrary results. Palkovicova et al. [56] demonstrated higher ammonia emissions in winter than in summer. Gallmann [51] also referred to various authors who were able to determine higher emission rates at lower temperatures. For CFD_S_, an increase of the ammonia emissions could not be found in this study, despite significantly higher ventilation rates. In accordance with the named authors, lower ammonia concentrations were measured for CFD_S_ compared with CD and CFD_W_. As shown in Section 2.3.1, the emission rates are calculated by the difference of fresh- and exhaust air concentrations. Due to the position of Barn I on the farm, the fresh air was already contaminated with higher ammonia concentrations than measured for Barn II and III. The fact that significantly lower exhaust air concentrations were measured in Barn I during CFD_S_ suggests that less ammonia must have been released in the barn under experimental conditions. Therefore, lower emissions could be explained by this, although higher ventilation rates were measured. Philippe et al. [22] achieved 50% reduction in ammonia emissions compared to a control group by use of crude fiber in fattening pigs. Other authors reached reductions of 30%–40% under laboratory conditions [57,58]. The average emissions in weight section 70–110 kg were approximately identical in CD and CFD_W_. Reduction of ammonia emissions during CFD_S_ correspond to those of the mentioned studies. However, a comparison of the summer and winter periods is only conditionally possible due to the different seasons and different barns. The fact that emissions during CFD_S_ were lower than values reported in other studies suggests that the crude fiber supplemented feeding had a reducing effect on ammonia emissions. Since the study was carried out on a farm under practical conditions, this factor must be taken into account. In comparison to Philippe et al. [22], this study investigated large group housing with differences in husbandry and management. This may have influenced the results and may have led to a lower reduction in ammonia emissions. It must be noted that the different diets were offered simultaneously in the different feeding areas. No precise conclusion can be made about the potential of ammonia reduction for the different crude fiber contents in the diet.

About 44% of ammonia emissions from fattening pig barns originate from polluted surface area [59]. Polluted surface area and ammonia emissions are directly related [5]. As shown by Massé et al. [58], increased crude fiber content in the diet can lead to an increase in fecal mass and a higher viscosity of manure. Both factors can result in an increased contamination of the surface area. In the presented study, no significant differences in surface contamination and occlusion of slats could be found between CD and CFD. On average, the amount of “clean and dry” surface area was around 44% for both the control and experimental group. Moreover, the amounts of “polluted” area in the different categories are comparable. More than half of the slats were not occluded by excrements for CD and CFD, respectively. As shown by the results, feed intake decreases by increasing crude fiber content in the diet. The fact that there was no significant difference in surface contamination could be explained by avoiding luxury consumption. Thus, it would be possible that the effects described by Massé et al. [60] are balanced with those of reduced feed intake. The results suggest that the increased use of crude fiber has no negative impact on floor cleanliness or ammonia emissions. Further, with regard to animal welfare, this is a positive factor, since negative effects—e.g., on claw health due to increased pollution [36,38]—cannot be assumed. For the same aspect, it is also positive that clear functional areas could be identified during the evaluation. It corresponds to the natural behavior of pigs [5]. This behavior is supported by the use of the presented feeding technology and the large group housing. The influence of feeding rich in crude fiber on the behavior of the animals was not subject of these studies. This aspect could be considered in subsequent studies in order to enable an evaluation of the improvement of animal welfare.

## 5. Conclusions

In summary, it can be said that the use of WPSs with a high crude fiber content increases the feeling of satiety and has an influence on feed intake. Different “types” of pigs showed differences in their feed intake capacity, especially the control of feed intake for more fat-prone animals, which was herein confirmed via WPSs. With this knowledge, a further development of the presented resource-efficient feeding concept is desirable to feed fattening pigs individually and according to their nutrient requirements. This will enable a reduction of luxury consumption and help save important resources. The next step is to take into account the new knowledge of the whole plant silage in terms of feed intake and to adapt the feed ingredients to each species, taking into account their average feed intake. Ammonia emissions were not negatively affected in this study. During the winter period, no deterioration in ammonia emissions compared to the control was observed. Ammonia emissions could be reduced in some sections. In summer, significantly lower ammonia emissions were found in the experimental group (also in comparison with values reported in other studies). Thus, a positive conclusion can be drawn in this regard. Moreover, no deterioration of floor cleanliness could be determined due to increased crude fiber content in the diets. This can be seen as a positive aspect, regarding the ammonia release as well as animal welfare. More research and investigations on this topic are currently being conducted. These will be necessary for a final overall evaluation of the resource-efficient feeding concept.

## Figures and Tables

**Table 1:** Chemical composition of “CF1”, “SF1”, “SF2”, and “Soybean oil”, according to the declaration (88% dry matter content; DM). Triticale WPS and the Corn-Cob-Mix (CCM) were analyzed in the Institute for Animal Nutrition Hanover (88% DM) [34].

Ingredients	CF1 (%)	SF1 (%)	SF2 (%)	Soybean Oil (%)	Triticale- WPS (%)	CCM (%)
Crude protein	16.00	25.00	21.50	0.00	7.10	9.12
Crude fat	3.50	3.50	3.00	97.00	1.76	4.11
Crude fiber	4.50	7.00	7.00	0.00	20.50	1.28
Crude ash	4.50	9.00	7.50	0.00	4.51	1.35
**Starch**	37.84	22.52	21.47	0.00	23.44	60.54
**Sugar**	4.36	6.04	5.17	0.00	1.33	11.00
Lysine	1.10	2.20	1.40	0.00	0.21	0.25
Methionine	0.32	0.35	0.40	0.00	0.07	0.20
Calcium	0.65	1.60	1.00	0.00	0.27	0.06
Phosphorus	0.45	0.60	0.65	0.00	0.20	0.31
Acid detergent fibre (ADF)	6.34	9.24	10.32	0.00	27.02	2.88
Metabolic energy (megajoule kg^−1^)	13.40	11.90	11.60	35.88	6.09	16.00

**Table 2:** Components given in Barn I and II to experimental groups fed at an average bodyweight of 70 kg.

Components	CF1 (%)	SF1 (%)	SF2 (%)	Soybean Oil (%)	Triticale-WPS (%)	CCM (%)
**Diet 1:**	0.0	0.0	52.5	0.0	2.5	45.0
**Diet 2:**	0.0	8.0	41.0	1.0	5.0	45.0
**Diet 3:**	0.0	18.8	27.1	1.6	7.5	45.0
**Diet 4:**	0.0	29.3	13.1	2.6	10.0	45.0

**Table 3:** Components given in Barn III to control group fed at an average bodyweight of 70 kg. Diet 5 was for the 50% lighter animals. Diet 6 was for the 50% of the heavier animals.

Components	CF1 (%)	SF1 (%)	SF2 (%)	Soybean Oil (%)	Triticale-WPS (%)	CCM (%)
**Diet 5:**	85.0	0.0	0.0	0.0	0.0	15.0
**Diet 6:**	90.0	0.0	0.0	0.0	0.0	10.0

**Table 4:** Calculated energy and nutrient contents per kg of dry matter in all diets for Barns I–III.

		Diet 1	Diet 2	Diet 3	Diet 4	Diet 5	Diet 6
ME	MJ kg DM	14.62	14.63	14.55	14.54	14.87	14.80
Ash	g/kg DM	43.15	44.85	47.80	50.38	44.85	46.55
Crude protein	g/kg DM	167.83	167.59	167.87	168.12	168.65	172.79
Crude fat	g/kg DM	37.18	47.05	53.33	63.21	27.97	27.14
Starch	g/kg DM	437.53	438.08	440.24	441.45	531.77	521.76
Sugar	g/kg DM	35.04	34.96	35.63	36.03	38.10	39.90
Crude fiber	g/kg DM	54.47	58.42	62.87	67.03	44.64	45.91
Lysin	g/kg DM	9.89	9.94	9.99	10.00	12.18	12.39

Diets 1–4 are compound diets, which were tested in the experimental groups. Diets 5 and 6 are the composed diets of the control group. An overview of the components used is shown in Table 2 and Table 3.

**Table 5:** Body composition and gender of the animals at the grouping time in Barn I.

Group	Ø	HF (n = 163)	HL (n = 165)	LF (n = 163)	LL (n = 164)
Body mass (kg)	49.82 ± 6.42	54.32 ^a^ ± 4.97	52.5 ^b^ ± 5.38	46.09 ^c^ ± 5.20	45.21 ^c^ ± 4.58
Male (%)	49.01	59.51	41.21	56.44	39.02
Female (%)	50.99	40.49	58.79	43.56	60.98
Backfat (cm)	0.69 ± 0.13	0.80 ^a^ ± 0.11	0.63 ^c^ ± 0.09	0.72 ^b^ ± 0.11	0.57 ^d^ ± 0.09
Muscle (cm)	3.50 ± 0.40	3.60 ^a^ ± 0.37	3.66 ^a^ ± 0.37	3.31 ^c^ ± 0.37	3.41 ^b^ ± 0.40
Backfat/Muscle	0.20 ± 0.03	0.22 ^a^ ± 0.02	0.17 ^b^ ± 0.02	0.28 ^a^ ± 0.02	0.17 ^b^ ± 0.02

^a,b,c,d^ averages differ significantly within a line (*p* < 0.05).

**Table 6:** Daily feed intake (in kg 88% DM) with a calculated body weight of 100 kg per animal in the four groups and diets.

	Ø	HF (n = 163)	HL (n = 165)	LF (n = 163)	LL (n = 164)
Diet 1	3.61 ± 0.65	3.65 ^A^ ± 0.67	3.88 ^A^ ± 0.93	3.74 ^AB^ ± 0.77	3.18 ^AB^ ± 0.79
Diet 2	3.51 ± 0.50	3.15 ^A^ ± 0.70	3.14 ^B^ ± 0.62	4.29 ^A^ ± 0.61	3.44 ^A^ ± 0.99
Diet 3	3.31 ± 0.44	3.60 ^A^ ± 0.32	2.75 ^B^ ± 0.70	3.89 ^AB^ ± 0.80	3.00 ^AB^ ± 0.52
Diet 4	3.20 ± 0.42	3.25 ^A^ ± 0.71	3.20 ^B^ ± 0.69	3.62 ^B^ ± 0.71	2.71 ^B^ ± 0.44

^A,B^ averages differ significantly within a column (*p* < 0.05).

**Table 7:** Climatic conditions during control and experimental feeding. Mean ± standard deviation.

Diet	Body Weight (kg)	Ventilation Rate (m^3^ h^−1^ LU^−1^)	Temperature Inside (°C)	Temperature Outside (°C)	Relative Humidity Inside (%)	Relative Humidity Outside (%)
CD	75–85	193.4 ^A^ ± 23.0	20.5 ^A^ ± 0.5	6.4 ^A^ ± 2.0	75.9 ^A^ ± 5.0	89.9 ^A^ ± 9.1
	105–115	177.3 ^D^ ± 28.8	18.7 ^D^ ± 0.9	2.4 ^D^ ± 2.4	80.5 ^D^ ± 6.3	91.4 ^D^ ± 8.4
	70–110	183.2 ^G^ ± 34.3	19.8 ^G^ ± 1.2	4.7 ^G^ ± 3.8	77.2 ^G^ ± 5.9	89.4 ^G^ ± 10.1
CFD_W_	75–85	193.2 ^B^ ± 59.5	19.5 ^B^ ± 0.8	6.4 ^A^ ± 3.6	71.5 ^B^ ± 6.5	79.7 ^B^ ± 14.5
	105–115	231.5 ^E^ ± 121.1	18.9 ^D^ ± 0.8	8.2 ^E^ ± 3.7	70.5 ^E^ ± 5.8	81.3 ^E^ ± 14.0
	70–110	204.7 ^H^ ± 62.2	19.2 ^G^ ± 0.9	7.2 ^H^ ± 3.3	71.7 ^H^ ± 6.1	81.4 ^H^ ± 14.0
CFD_S_	75–85	509.3 ^C^ ± 156.5	20.6 ^C^ ± 2.2	16.1 ^B^ ± 4.7	66.7 ^C^ ± 10.4	67.9 ^C^ ± 19.3
	105–115	457.6 ^F^ ± 33.8	24.1 ^E^ ± 4.1	21.9 ^F^ ± 6.6	60.7 ^F^ ± 12.0	55.1 ^F^ ± 19.2
	70–110	497.4 ^I^ ± 96.4	23.3 ^H^ ± 3.9	20.2 ^I^ ± 6.4	65.2 ^I^ ± 12.2	62.9 ^I^ ± 21.5

CD: Control diet; CFD_W_: Crude fiber diet (winter); CFD_S_: Crude fiber diet (summer); LU: Livestock unit, equal to 500 kg body weight. ^A;B;C^ values differ significantly in weight section 75–85 kg within a column (*p* < 0.05). ^D;E;F^ values differ significantly in weight section 105–115 kg within a column (*p* < 0.05). ^G;H;I^ values differ significantly in weight section 70–110 kg within a column (*p* < 0.05).

## Supplementary Materials

The following are available online at https://www.mdpi.com/2076-2615/10/3/497/s1, Table S1: Components given in Barn I to experimental groups; fed at an average bodyweight of 50 kg, Table S2: Components given in Barn I to experimental groups; fed at an average bodyweight of 70 & 90 kg, Figure S1: Average Evaluation of score areas in Barn I and III and Illustration of the different functional areas.
