# Supplementary material for: Validation of a New Resource-Efficient Feeding System for Fattening Pigs Using Increased Crude Fiber Concentrations in Diets: Feed Intake and Ammonia Emissions
Source: Animals (Basel). 2020 Mar 16;10(3):497. doi: 10.3390/ani10030497 (PMC7142619; doi:10.3390/ani10030497)
Supplement: Supplementary file 1 [file animals-10-00497-s001.pdf]

Diets & Components given in Barn I to experimental groups

Table Supplementary 1. Components given in Barn I to experimental groups; fed at an average bodyweight of 50 kg

Components	CF1	SF1	SF2	Soybeanoil	Triticale WPS	CCM
Diet 1	39,65	27,85	0,00	0,00	2,5	30,0
Diet 2	32,93	30,94	0,00	1,13	5,0	30,0
Diet 3	25,33	34,86	0,00	2,31	7,5	30,0
Diet 4	17,73	38,78	0,00	3,50	10,0	30,0

		Diet 1	Diet 2	Diet 3	Diet 4
ME	MJ kg DM	14,64	14,60	14,55	14,51
ME	MJ kg FM	12,88	12,85	12,81	12,77
ash	g/kg DM	52,53	53,84	55,59	57,33
crude protein	g/kg DM	181,17	180,34	180,34	180,35
crude fat	g/kg DM	38,42	48,98	60,10	71,22
starch	g/kg DM	454,81	440,46	424,44	408,42
sugar	g/kg DM	44,32	43,66	43,17	42,68
crude fibre	g/kg DM	54,12	59,23	64,58	69,93
ADFom	g/kg DM	70,42	76,97	83,79	90,62
Lysin	g/kg DM	11,40	11,52	11,64	11,76

Table Supplementary 2. Components given in Barn I to experimental groups; fed at an average bodyweight of 70 & 90 kg

	CF1	SF1	SF 2	Soybeanoil	Triticale WPS	CCM
**Diet 1**	0,00	0	52,5	0	2,5	45
**Diet 2**	0,00	8	41	1	5	45
**Diet 3**	0,00	18,8	27,1	1,6	7,5	45
**Diet 4**	0,00	29,28	13,14	2,58	10	45

		Diet 1	Diet 2	Diet 3	Diet 4
ME	MJ kg DM	14,62	14,63	14,55	14,54
ME	MJ kg FM	12,87	12,87	12,80	12,80
ash	g/kg DM	43,15	44,85	47,80	50,38
crude protein	g/kg DM	167,83	166,59	167,87	168,12
crude fat	g/kg DM	37,18	47,05	53,33	63,21
starch	g/kg DM	437,53	438,08	440,24	441,45
sugar	g/kg DM	35,04	34,96	35,63	36,03
crude fibre	g/kg DM	54,47	58,42	62,87	67,03
ADFom	g/kg DM	80,85	83,61	86,49	88,98
Lysin	g/kg DM	9,89	9,94	9,99	10,00

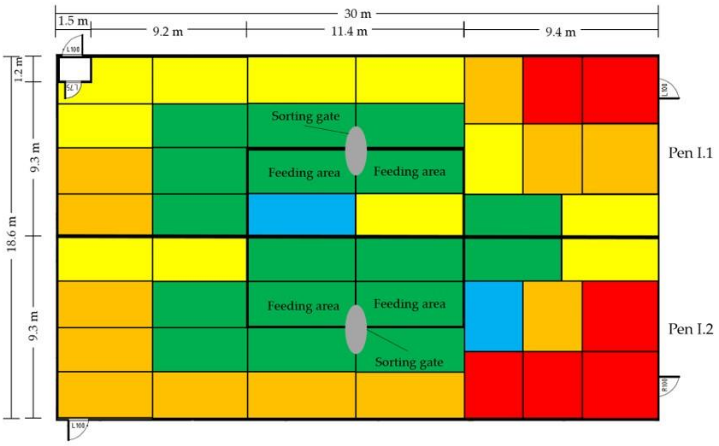

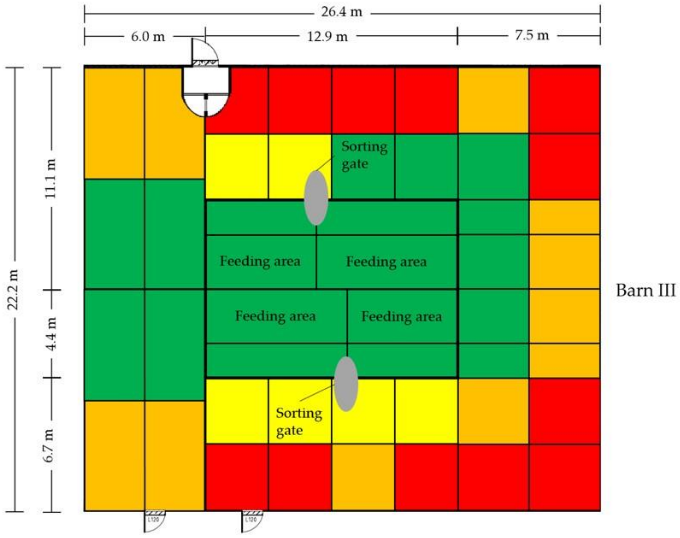

Figure 1 Supplements.jpg
